# Dust

**Published:** 1919-10-04

**Authors:** 


### DUST.

### By a SOCIAL WORKER.

The present writer, recovering tediously from an illness caused by dust, thinks it will be well if more attention is drawn to its mischief. The fact that "Dust thou art, to dust returnest" is no mitigation of the injury done to man's exquisitely framed mechanism by the assaults of dust in its dry form. Many have had or are having a similar experience in a year when local authorities who seek to economise, defer as late as they can without rousing too great wrath the procession of the water-cart. First a tired physique, after the long winter, then a slight chill, a sore throat. Then a week of unresting north-east wind in a town of which the soil powders readily into white *tourbillons*, which filled every street from morn till an eve that was not dewy. Indoors white dust settled grittily on book or writing-pad, clothing gathered it, the more readily if one chanced to be caught in an occasional flurry of sleet which damped the fabric. Ultimate result, a weakened power of resistance and a sore patch of throat, upon which a wandering zymotic germ found hospitality and ran its course. "Curious how common these cases are this year," observes the doctor, who is hurried, poor man; there is such a lot of illness in the poorer streets, and his partner is not yet demobilised. When the water-carts do come out it will not be in those poorer streets that they will do most work. A working woman, whose attention had been called to hygienic questions through joining a Women Citizens' Association, related last summer that she took her children one afternoon to a London park and watched the repeated soaking of the roadway there, while her own street had been untouched all day.

The Dust of Slums.

Yet the dust manufactured in the slum cannot be shut up there. In London a north-east wind will gather tuberculous and zymotic bacilli as it passes through East Ham and sweeps on into Belgravia. Even the health teachers have done little to warn the working-class housewife against the dust-devil. Rather the contrary. "Have loose squares of carpet and mats that can be easily shaken out of doors." They can, indeed! Come in through the suburbs of town by an early train, and watch the hundreds of pairs of arms at work with rolled-up sleeves in tiny back yards, producing amazing results with quite a small hearthrug each. In the poorer districts, by the way, these women will very probably be wearing a man's old cloth cap, which covers their hair. It is curious that the one argument for the housemaid's cap, which one seldom or never hears raised, is that when of comfortable size it protected the hair from dust while the wearer made beds and swept. When reduced to a minute frill poised on the chignon its practical use had so disappeared that the hygienic argument is lost.

A Natural Instinct.

It is, after all, a natural instinct in the working woman, against which the hygienists have fought hard on other grounds, which makes her keep the windows of her best parlour, and even of bedrooms, raised so little above the dusty street, severely closed. And it is time, surely, that health authorities interfered with the builder alike of costly and of cheap house, and with the makers of furniture. What spirit of mischief inspired man with the invention of the ceiling cornice? Well out of sight, and probably therefore out of mind, in every bedroom of every "good" house, a neatly contrived shelf for the storage of dust runs round the ceiling, possibly, indeed, there are several such, and floriated ornaments to aggravate the mischief. And the same mischievous goblin must have whispered to the inventor of the first wardrobe that a hollow space on the top, fenced off by an ornamental coping so shaped as to make it difficult for a woman's arm to clean out the enclosure, would admirably suit the same purpose. While it would be a further gain to stand the furniture upon legs too short to allow of brushing underneath, or on a frame having three sides, so that a woman should risk straining herself by moving the wardrobe out, or allow dust to gather. Gas stoves are now recommended as "so clean," but are almost always so fixed that to remove dust from under and behind them is almost impossible.

Money and Working Women.

We must thankfully acknowledge, however, that the increase in money available for working women has resulted in much buying of furniture, which has enabled many a tidy soul to clear the rubbish of cardboard boxes and bundles out from under beds and in corners with great gain in freedom from gatherings of dust, as well as in her personal comfort and convenience.

Function of School Lessons.

Probably much good will be done if more stress is laid in school lessons on the real dangers to throat and lungs, and by way of infection, of dust in houses and streets. The children can easily learn that the way to cope with dust is not to flick it off into the air or on to the floor. Irish girls, accustomed to earthen floors, and French ones, used to boards, will always do this on entering domestic service. They will also try to clean a room by raising with furious broom-work a storm of dust and carpet-fluff, which will not finish settling for very many hours. All machinery which will gather dust from a soft fabric, or extract it without wearing away the pile of woollen materials needs to be encouraged. It is said that analysis of the air on the summit of high Alps has shown that even there a trifling amount of dust is held in the atmosphere, though so little that irritated throats and lungs find natural healing. It is certain that we can considerably diminish preventable disease and ill-health by attention to this enemy within our gates.

